# The Neurovascular Unit Dysfunction in the Molecular Mechanisms of Epileptogenesis and Targeted Therapy

**DOI:** 10.1007/s12264-024-01193-3

**Published:** 2024-03-30

**Authors:** Xiuxiu Liu, Ying Zhang, Yanming Zhao, Qian Zhang, Feng Han

**Affiliations:** 1 Medical Basic Research Innovation Center for Cardiovascular and Cerebrovascular Diseases, Ministry of Education, Nanjing, 211166 China; 2 International Joint Laboratory for Drug Target of Critical Illnesses, School of Pharmacy, Nanjing Medical University, Nanjing, 211166 China; 3 Institute of Brain Science, The Affiliated Brain Hospital of Nanjing Medical University, Nanjing, 211166 China; 4 Gusu School, Nanjing Medical University, Suzhou Municipal Hospital, The Affiliated Suzhou Hospital of Nanjing Medical University, Suzhou, 210019 China

**Keywords:** Epileptogenesis, Neurovascular unit, Molecular mechanisms, Drug targets

## Abstract

Epilepsy is a multifaceted neurological syndrome characterized by recurrent, spontaneous, and synchronous seizures. The pathogenesis of epilepsy, known as epileptogenesis, involves intricate changes in neurons, neuroglia, and endothelium, leading to structural and functional disorders within neurovascular units and culminating in the development of spontaneous epilepsy. Although current research on epilepsy treatments primarily centers around anti-seizure drugs, it is imperative to seek effective interventions capable of disrupting epileptogenesis. To this end, a comprehensive exploration of the changes and the molecular mechanisms underlying epileptogenesis holds the promise of identifying vital biomarkers for accurate diagnosis and potential therapeutic targets. Emphasizing early diagnosis and timely intervention is paramount, as it stands to significantly improve patient prognosis and alleviate the socioeconomic burden. In this review, we highlight the changes and molecular mechanisms of the neurovascular unit in epileptogenesis and provide a theoretical basis for identifying biomarkers and drug targets.

## Introduction

Epilepsy, one of the prevalent neurological disorders, is characterized by spontaneous and recurrent unprovoked seizures, affecting ~1% of the global population [1]. Although clinical practice offers a wide array of anti-epileptic drugs, their efficacy is limited to seizure control, with a notable absence of medications capable of averting the onset of epilepsy [2]. Moreover, a significant proportion, ~30%–40% of epilepsy patients experience drug resistance [3]. Therefore, it is of utmost importance to investigate the mechanisms underlying epileptogenesis and identify potential drug targets for preventing seizures.

Epileptogenesis, the process of structural and functional changes in the brain following potential epileptogenic lesions [4], plays a crucial role in the development of epileptic seizures. The neurovascular unit (NVU), composed of astrocytes, microglia, neurons, vascular endothelial cells, vascular pericytes, and extracellular matrix, is central to epileptogenesis [5, 6]. Disorders within the NVU involve an imbalance of the neuronal excitation-inhibition system, the morphology and functional alteration of astrocytes, inflammatory activation of microglia, and increased activity of vascular endothelial cells [7]. Considering that it takes months to years from initial brain damage to final seizures [8], long-term follow-up of the affected population is impractical. Therefore, the identification of reliable biomarkers during the epileptogenesis process is crucial in identifying the patients undergoing epileptogenesis and selecting effective anti-epileptogenic drugs for treatment [9]. This endeavor has important implications for delaying the progression of epilepsy and preventing seizures, especially in cases of drug-resistant epilepsy [10]. However, current research on epilepsy predominantly focuses on seizures, with limited study of epileptogenesis. Hence, in this review, we aim to comprehensively summarize the molecular mechanisms and biomarkers underlying the involvement of the NVU in epileptogenesis, along with exploring therapeutic agents implicated in this process. By shedding light on this critical aspect of epilepsy, we hope to pave the way for more effective treatments and improved outcomes for patients facing this challenging condition.

## Changes of the Neurovascular Unit in Epileptogenesis

Reduced metabolic activity is a prominent feature underlying the early stages of epileptogenesis [11]. In the model of spontaneous recurrent seizures, a correlation has been established between decreased metabolic activity in early epileptogenesis and the parameters of seizure latency, duration, and frequency. This correlation has been observed through positron emission tomography imaging with the radiolabeled glucose analog fluorodeoxyglucose [2]. Brain homeostasis and metabolic function can be considered as an interaction between neurons, glia, and cerebral circulation [12]. Thus, any functional alterations or disturbances within these components can significantly influence brain metabolic activity. Understanding these intricate interactions is crucial for comprehending the mechanisms during early epileptogenesis and may ultimately contribute to the development of more effective interventions to address epilepsy in its early stages.

### Neuron in Epileptogenesis

Changes in the levels of excitatory and inhibitory transmitters and ion channels have been identified as critical neuronal mechanisms involved in epileptogenesis [13, 14]. Due to the heightened sensitivity of neurons to external changes, various potential factors can induce neuronal hyperexcitability, leading to the development of epileptic seizures [15, 16]. Notably, there exists considerable neuronal heterogeneity between epileptogenic and non-epileptogenic tissues, particularly in terms of population metabolic activation. Neuronal heterogeneity may play a crucial role in determining the brain’s resilience to transient changes when neural circuits transit into a synchronized state. Networks characterized by high heterogeneity exhibit a stable state of asynchronous firing activity and can better adapt to transient shifts towards a more active and synchronized state. Conversely, networks with low heterogeneity play a crucial role in mediating synchronous discharges in epilepsy [17]. Understanding these complexities of neuronal heterogeneity is important for unraveling the underlying mechanisms of epileptogenesis and may provide valuable insights into the development of targeted therapies for epilepsy treatment.

### Astrocytes and Microglia in Epileptogenesis

The epileptic brain experiences structural changes in astrocytes. In a randomized controlled trial, S100β levels in the serum were increased in epileptic patients, suggestive of reactive astrocytes [18]. Reactive astrocytes can exhibit at least two distinct activation states: A1 and A2, respectively exhibiting anti-inflammatory neuroprotective and pro-inflammatory neurotoxic effects [19]. The proliferation of astrocytes can facilitate the formation of glial scarring and promote axon regeneration within the CNS, potentially offering benefits [20]. Nonetheless, gap junction-mediated abnormal synchronous neuronal firing is a significant characteristic of epileptic events [21]. Astrocyte activation can lead to an increase in gap junction coupling, rendering the number of electrical synapses and electrical conductivity increased, which in turn promotes the expansion of synchronous neuronal firing—a key characteristic of epileptic events [22]. Therefore, gap junction dysfunction may serve as a potential mechanism underlying epileptogenesis. In addition, the function of astrocytes is altered in epilepsy. Astrocytes can synthesize glutamine by glutamine synthetase (GS), which is involved in the synthesis of glutamate transmitters [23]. Deficiency of *GS* is reported in astrocytes in the human epileptic brain and knockout of the *GS* gene in mice can increase susceptibility to epilepsy [24]. Changes in the function of GS in astrocytes ultimately alter the levels of glutamate transmitters and affect excitatory transmission in the brain.

After traumatic brain injury (TBI) or other seizures-inducing stimuli, microglia undergo rapid activation and can remain in this state for extended periods, ranging from months to even years [25, 26]. Once activated, microglia polarize into distinct phenotypes and subsequently release various pro-inflammatory or anti-inflammatory mediators [27, 28]. These microglia-mediated responses have implications for brain repair processes and play a differential role in the development of post-traumatic epilepsy [29]. With microglial polarization into the neuroprotective phenotype, they exhibit a neuroprotective effect mediated through the platelet P2Y12 receptors (P2Y12Rs) and resolve the inflammation by promoting the repair of damaged tissue through matrix remodeling [30, 31]. In addition, microglia contribute to the phagocytosis of excess new granule cells in the dentate gyrus, reducing the formation of aberrant neural circuits and maintaining the homeostasis of the dentate circuitry [32]. Conversely, when microglia polarize into neurotoxic phenotypes, they release cytokines that promote neuroinflammation and consequently contribute to epileptogenesis. Furthermore, microglia can induce neuronal hyperexcitability [33, 34]. Single-cell transcriptomics in human brain epilepsy has also found that microglia exhibit a proinflammatory phenotype and increased expression of proinflammatory cytokines and chemokines in refractory epilepsy [35]. They also lead to the death of certain neurons and oligodendrocytes and decrease the number and strength of synapses [36]. Fully understanding the morphology, function, and pathological processes of astrocytes and microglia will aid in the discovery of pathological mechanisms in epileptogenesis.

### Vascular Endothelial Cells in Epileptogenesis

Enhanced expression of vascular endothelial growth factor (VEGF) has been reported in hippocampal slices from patients with drug-resistant epilepsy [37]. Consistently, reduced cerebral blood flow and tissue hypoxia can trigger an increased expression of VEGF in vascular endothelial cells, thus inducing abnormal angiogenesis and increasing microvascular structural density during epileptogenesis [38]. It is suggested that this adaptative process may improve perfusion and meet the heightened metabolic demands of neurons. However, sustained increased expression of VEGF can disrupt tight junctions and activate matrix metalloproteinase that degrades the blood vessel wall, ultimately increasing vascular permeability and leading to chronic damage to the blood-brain barrier (BBB) [39], whose dysfunction has been suggested to play an important role in epilepsy. However, the mechanism mediating the transition from cerebrovascular damage to epilepsy remains unclear.

## Molecular Mechanisms and Potential Drug Targets During Epileptogenesis

**Fig. 1:**
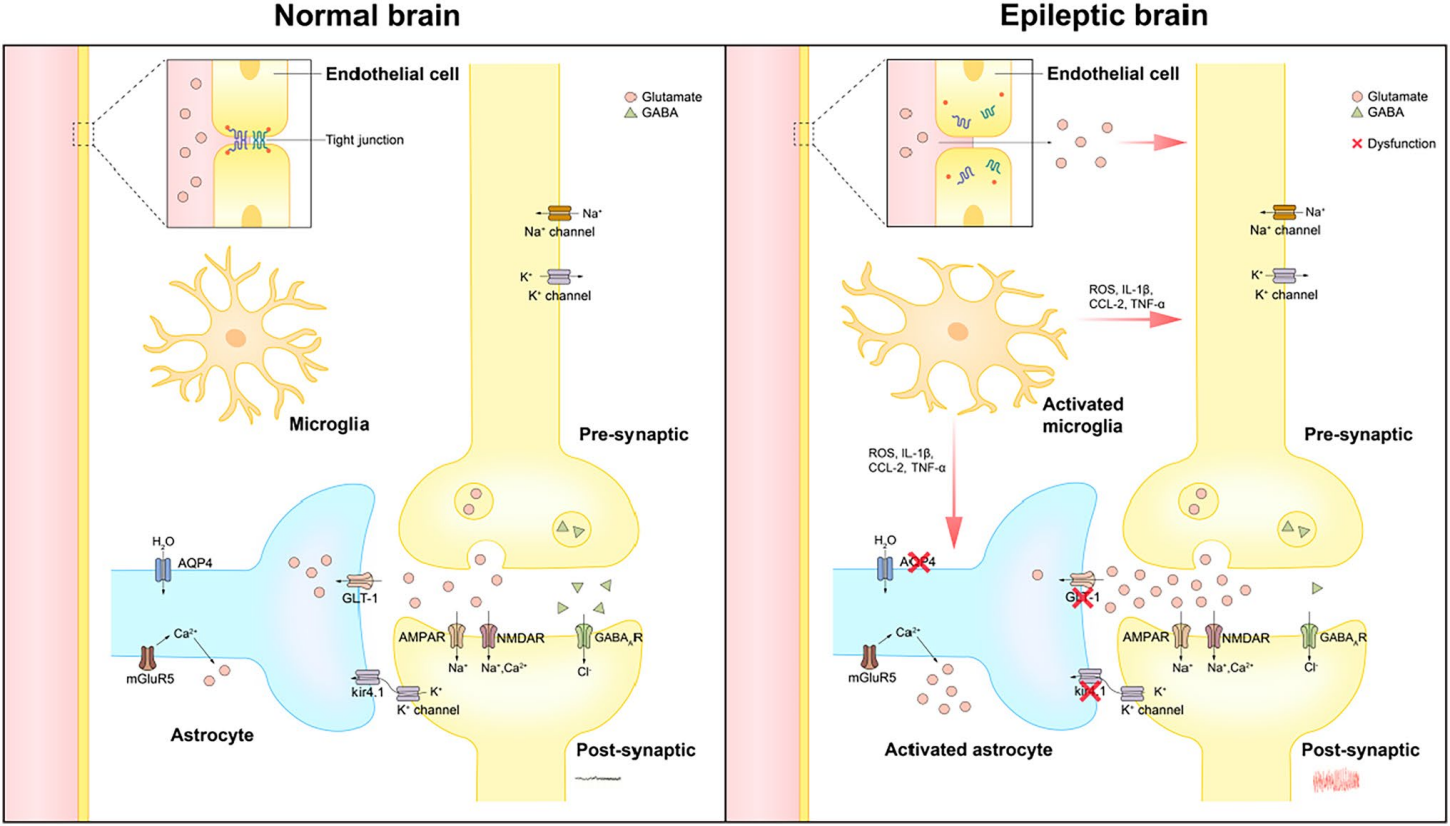
The molecular mechanism of NVU disorder involved in mediating the epileptogenesis.

The NVU is an intricate and specialized structure unique to the CNS, comprising multiple cell types that function together [40]. Under pathological conditions, disruptions to the precise cellular and homeostatic interactions within the NVU can induce abnormal neuronal firing or seizures [41]. Understanding the molecular mechanism underlying NVU disorders, which play a critical role in mediating epileptogenesis, holds great potential in identifying drug targets during the occurrence of epilepsy (Fig. 1).

### Disturbance of the Neuronal Excitation-Inhibition System

The prevailing understanding is that epileptogenesis arises from an imbalance in neuronal excitation-inhibition [42], where an increase in excitatory effects and a decrease in inhibitory effects leads to neuronal hyperexcitability and the occurrence of epileptic seizures [43].

In the CNS, γ-aminobutyric acid (GABA) serves as an inhibitory neurotransmitter that binds to GABA_A_ receptors, responsible for transmitting most of the inhibitory signals in the brain [44]. This binding induces hyperpolarization of neuronal cell membranes, resulting in inhibitory effects. In pathological conditions, including TBI, GABAergic interneurons may become impaired, leading to a reduction in the expression of the GABA_A_ receptor-stabilizing protein ubiquitin1 (Ubqln1). The decrease in Ubqln1 and impaired GABAergic interneurons result in reduced inhibitory effects of GABAergic interneurons, leading to increased neuronal excitability and heightened susceptibility to epilepsy [45]. In an *in vitro* epilepsy model, researchers have found that using niacinamide (NM) to increase Ubqln1 expression can stabilize GABA_A_ receptors to relieve the generation of epileptiform activity [46], suggesting that modulating Ubqln1 could be a potential strategy for alleviating the epileptogenesis.

Glutamate, the primary excitatory neurotransmitter in the brain, causes depolarization of the postsynaptic membrane of neurons and mediates rapid excitatory synaptic neurotransmission in the CNS by binding to AMPA and NMDA receptors. However, under pathological conditions, the excessive accumulation of glutamate in synapses can lead to neuronal hyperexcitability and disrupt the excitation-inhibition balance, contributing to the development of epileptogenesis [47]. Studies have indicated that increased levels of glycogen synthase kinase 3β, which regulates neuronal transmission and plasticity, can inhibit the phosphorylation of the GluA1 subunit of AMPA receptor. This inhibition can protect the neuronal network and slow down the progression of kainic acid (KA)-induced epileptogenesis [48].

The combination of excitatory or inhibitory transmitters with their corresponding receptors leads to ion channel activation, which plays an important role in epileptogenesis. Loss of function of NaV1.1 reduces electrical excitability of GABAergic neurons [49]; mutation of the α subunit of CaV2.1 suppresses neurotransmitter release mediated by the pre-synaptic neuronal membrane action potential [50]; and inactivation of KV1.2 leads to increased neurotransmitter release from excitatory neurons [51]. These ion channel changes affect the membrane potential and neurotransmitter release of neurons, ultimately triggering hypersynchronous firing and epileptogenesis.

Traditionally, neurotransmitters and ion channels have been regarded as classical drug targets for epilepsy treatment. However, preventing or postponing seizures at the stage of epileptogenesis through targeting these components requires exhaustive research.

### Imbalance of Astrocyte Water and Ion Homeostasis

Astrocytes are crucial for maintaining ion homeostasis in the CNS and aiding tissue repair [52]. Nevertheless, under pathological conditions, changes in water channel and ion channel function on astrocytes disrupt water and ion homeostasis, contributing to epileptogenesis.

Aquaporin 4 (AQP4) is the most abundantly expressed aquaporin in astrocytes and is highly enriched at their end feet [53]. As it facilitates bidirectional water transport in response to osmotic gradients, AQP4 is critical for maintaining water homeostasis. Dysfunction of AQP4 causes changes in the extracellular space (ECS) and osmotic pressure [54]. The reduction of ESC increases extracellular ion concentration and strengthens tactile interactions between neurons, leading to more synchronous firing. Neurons are extremely sensitive to changes in ESC and osmotic pressure, and such abnormal conditions can lead to neuronal damage [55]. Consistently, an increased number of spontaneous seizures and prolonged duration of seizures are observed in *AQP4* knockout mice [56]. Moreover, the reduction and redistribution of AQP4 at the astrocytes' end feet are also a contributing mechanism to epileptogenesis [57].

Astrocytes use an inward rectifier K channel, particularly Kir4.1, to transfer K^+^ from regions of high concentration to low concentration [58], crucial for maintaining resting membrane potential and K^+^ homeostasis. Kir4.1 allows both inward and outward flow of K^+^, with a preference for inward flow. Knockdown of *Kir4.1* depolarizes the astrocyte resting membrane potential and impairs the ability to transfer K^+^ and glutamate into the cell [59], causing extracellular glutamate accumulation and eventually inducing seizures [60].

The Ca^2+^ signaling in astrocytes is primarily activated through Gq protein-coupled receptors (GqPCRs), followed by the release of Ca^2+^ from the endoplasmic reticulum *via* IP3R2. Metabotropic glutamate receptor 5 (mGluR5), a prominent GqPCR in astrocytes, can induce the release of Ca^2+^-dependent glutamate when upregulated [61]. The increase and accumulation of extracellular glutamate can cause excessive excitation of peripheral neurons and promote the development of epilepsy.

As part of the Na^+^-dependent transporter family, glutamate transporter 1 (GLT-1) is mainly expressed in astrocytes and accounts for >90% of total glutamate clearance. Changes in GLT-1 expression significantly affect inter-synaptic activity and the maintenance of extracellular glutamate homeostasis as well as glutamate-glutamine cycling [62]. In the KA-induced seizure model, GLT-1 is significantly up-regulated in the ipsilateral dorsal hippocampus at 1 and 3 days, but its expression is notably down-regulated at 7 days, coinciding with the occurrence of epileptic seizures [63]. Dysfunction of GLT-1 hinders astrocytes’ ability to effectively remove synaptic glutamate, leading to its accumulation and causing neuronal excitotoxicity, thereby contributing significantly to epileptogenesis.

Channels such as AQP4, Kir4.1, and GLT-1 are highly expressed in astrocytes. Drugs targeting aqueous or ion channels in astrocytes have the potential to inhibit their abnormal activation, reduce damage to peripheral neurons, and play an important role in anti-epileptogenesis.

### Inflammatory Activation of Microglia

Microglia serve as the immune cells of the CNS, responsible for removing cellular debris and releasing cytokines, which is essential for maintaining brain homeostasis [64]. Microglia play dual roles in epileptogenesis, namely, anti-epileptic and pro-epileptic effects. On the one hand, it is also noted above that microglia can regulate neural activity and protect neurons through mechanisms such as P2Y12Rs, mechanism remodeling, and excessive phagocytosis of new cells. In addition, Chen *et al.* found that *Trpm 2* knockout in microglia can regulate autophagy and reduce inflammation through recruiting the AMPK/mTOR pathway [65]. On the other hand, microglia also play a non-negligible role in promoting epilepsy. In response to brain damage, microglia undergo acute activation, transforming into the M1 phenotype that releases proinflammatory factors such as IL-1β, CCL2, CXCL9, and ROS, ultimately causing neuroinflammation and inducing pro-epileptic effects [66]. In a unilateral endocortical KA mouse model, Henning *et al*. found that reactive microglia are the major producer of TNF-α during early epileptogenesis and contribute to status epilepticus [67]. The inflammatory factors released by microglia can also trigger abnormal activation of astrocytes, causing reactive astrogliosis, which plays a significant role in epileptogenesis [68]. In addition, the involvement of microglia in the removal or formation of synapses can also contribute to epilepsy [69]. The abnormal pruning of synapses by microglia leads to a synaptic excitation-inhibition imbalance, which is a known cause of seizures. Certain mutations, such as program protein mutations, can cause microglia-specific phagocytosis of inhibitory synapses, eventually promoting epileptogenesis [70].

### Aberrant Activation of Vascular Endothelial Cell

Vascular endothelial cells not only compose the inner wall of blood vessels but also participate in essential functions, such as phagocytosis of necrotic or aging tissue and engagement in immune activity [71]. Disorders of the vascular system can contribute to the progression of nervous system diseases. As part of the BBB, tightly arranged vascular endothelial cells separate the blood from the brain’s extracellular fluid, restricting the passage of hydrophilic molecules and macromolecules [72]. In the event of brain damage, the generated inflammatory factors [73] and VEGF [74] induce a decrease in the tight junction protein of endothelial cells and an increase in BBB permeability. Consequently, an excessive amount of glutamate is transmitted into the brain, triggering abnormal neuronal activity and ultimately contributing to epileptogenesis [75]. Furthermore, apart from causing BBB damage, vascular endothelial cells can also promote epileptogenesis through vascular endothelial cell-derived risk factors. Studies have shown that the knockdown of cyclin-dependent protein 5 (*CDK5*) in endothelial cells increases the expression of chemokine ligands 1 protein (CXCL1) released by these cells. CXCL1 acts on astrocytes and disrupts GLT-1 function, leading to an increase in synaptic glutamate transmitter and, ultimately, epileptogenesis [76].

## Biomarkers of Epileptogenesis

**Fig. 2:**
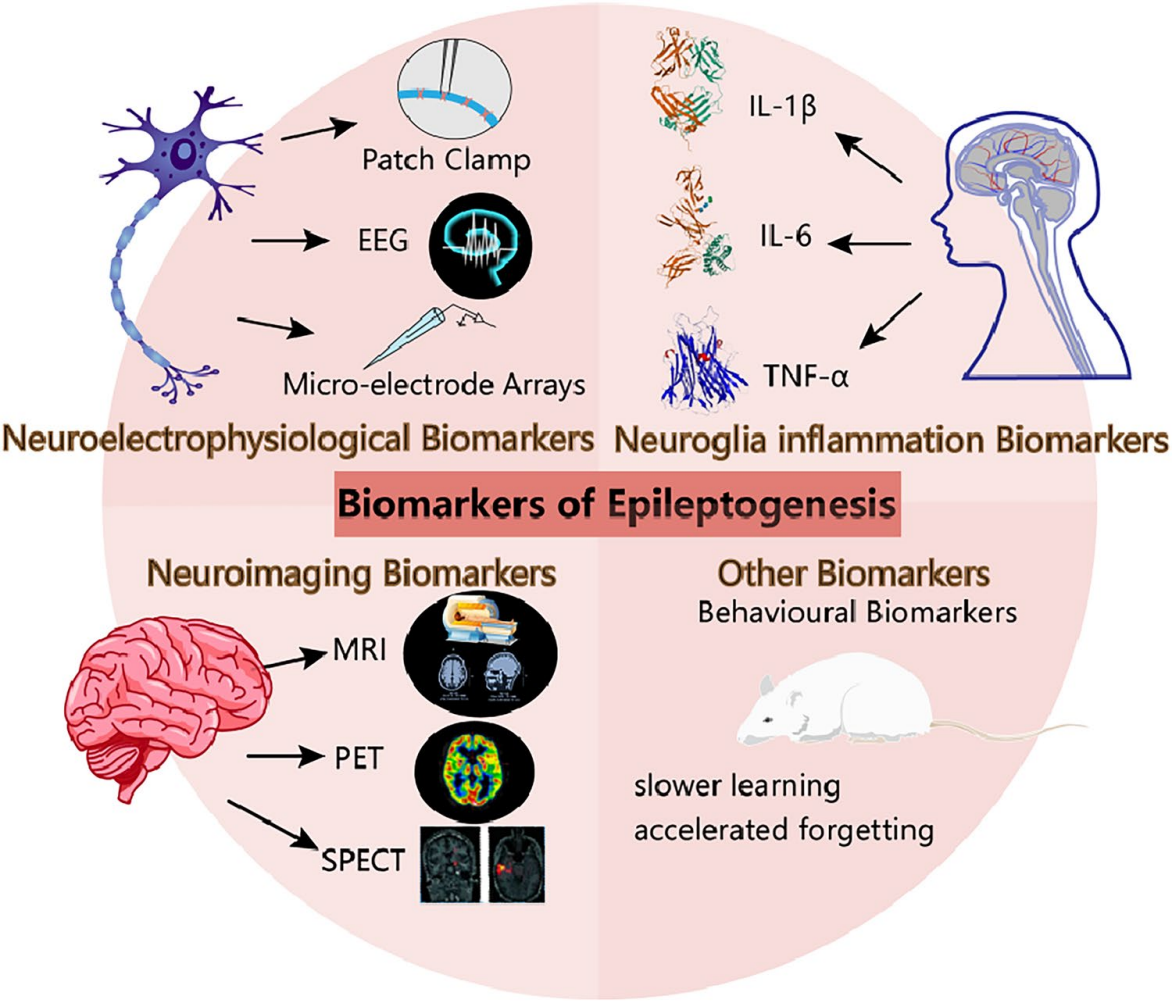
The potential biomarkers of epilepsy include molecular biomarkers, neuro-electrophysiological biomarkers, and neuroimaging biomarkers.

Currently, drug treatments for epilepsy primarily aim to manage symptoms and prevent the occurrence of seizures. However, these medications cannot address the underlying causes or prevent the development of epilepsy following a brain injury or insult. As a result, there is a pressing need for research and development of preventive drugs that can modify the course of epileptogenesis and effectively intervene and mitigate the risk of epilepsy development. The key to successful preventive treatment lies in identifying individuals at risk before experiencing their first seizure through reliable biomarkers. Considering the involvement of NVU dysfunction in epileptogenesis, biomarkers can be broadly categorized into three types: neuro-electrophysiological biomarkers, neuroglial inflammation biomarkers, and neuroimaging biomarkers (Fig. 2). By applying these biomarkers, researchers could identify high-risk individuals and implement preventive treatments that target the underlying mechanisms of epileptogenesis. Ideal biomarkers should help predict and early intervene in seizures in susceptible individuals and improve their quality of life.

### Neuro-electrophysiological Biomarkers

In the field of neuroelectrophysiology, two essential techniques are employed to study neuronal activity during epileptogenesis: electroencephalography (EEG) and patch-clamp electrophysiology.

EEG records the activity of large neuronal firing populations at the level of field potentials during epileptogenesis [77]. EEG is a non-invasive and widely-used technique that can measure whole-brain neuronal activity in real-time [78], which can reveal changes in the frequency of background brain activity (α, β, δ, θ, and γ activity) [79], therefore helping clinicians identify seizure activity. Previous studies have investigated dimensional changes of nonlinear dynamics in EEG signals after various epileptic lesions and found that the reduction in the nonlinear dynamics dimension in EEG tracings during early epileptogenesis can serve as a sensitive prognostic biomarker. These findings indicate that the magnitude of the EEG decline is positively correlated with the severity of epilepsy [80].

On the other hand, patch-clamp electrophysiology provides information about single-cell neuronal activity by studying real-time current data generated by ion channels [81]. This technique allows researchers to assess neuronal activity at the microscopic level.

By combining EEG and patch-clamp electrophysiology, researchers can bridge the gap between macroscopic and microscopic scales of neuronal activity. The combination of the two techniques represents a new approach to assessing the balance between excitability and inhibition in the brain during epileptogenesis. Moreover, these techniques can potentially provide valuable biomarkers for the prediction, evaluation, and treatment of epilepsy.

Studies have found that post-injury epilepsy using theta band activity has high sensitivity and specificity, both exceeding 90%. The dynamic change in the theta wave is negatively correlated with epilepsy latency. Specifically, a steeper slope of the theta wave is associated with a significant reduction in latency [82]. Several days after KA-induced seizure（SE） in the rat hippocampus, high-frequency oscillations (HFOs) emerged only in animals that later developed epilepsy [83]. Therefore, the change of theta band and the appearance of HFOs during epileptogenesis can be used as potential biomarkers to diagnose epileptogenesis and predict epileptic seizures.

Emerging electrophysiological technology such as the multi-dimensional micro-electrode array holds promise in monitoring sudden brain activity with improved spatial resolution of EEG and can help to identify the neuroelectrophysiological biomarkers of epileptogenesis in the human brain [84]. Focal cortical dysplasia is a common cause of epileptic seizures. A linear multi-electrode array was utilized to record the extracellular electrical potential of the cortical and subcortical brain regions near the site of frozen lesions (FLs) in anesthetized mice. This study showed that neonatal FLs promote a hyperexcitatory pattern of burst activity induced by anesthesia and disrupt the field potential synchronization between cortical and subcortical regions near the site of cortical malformations [85].

The hyperpolarized (HCN) channel, depolarized Ca^2+^ channel, and sodium-potassium-chloride-one/potassium-chloride-two (NKCC1/KCC2) co-transporter are representative channels responsible for hyperpolarization, depolarization, and ion flow-through, respectively. These channels can serve as potential biomarkers to aid the evaluation of epileptogenesis using electrophysiology tools such as patch-clamp technology [86]. Changes in the number and function of these channels can lead to alterations in electrophysiological characteristics. HCN is typically associated with hyperpolarization and can lead to hyperexcitability when mutations disrupt its normal function [87]. The CaV channel, on the other hand, leads to various depolarization and intracellular activity variations when it undergoes mutations affecting its normal function [88]. The impaired ability of NKCC1/KCC2 cotransporters to maintain intracellular low Cl^-^ has been found on the condition that the expression ratio of NKCC1 surpasses that of KCC2, resulting in the accumulation of biophysical Cl^-^ currents within the cells, ultimately leading to pathological hyperexcitability [89]. The alterations in electrophysiological characteristics of these channels can lead to an imbalance between excitation and inhibition, and such changes can be captured by the patch-clamp technology. In conclusion, the hyperpolarized, depolarized, and flow-through ion channels can potentially serve as tri-coordinate biomarkers of electrophysiological dysfunction in the context of epileptogenesis.

### Biomarkers of Neuroglial Inflammation

Neuroinflammatory pathways in the brain play a significant role in epileptogenesis [66]. Both preclinical and human studies have provided compelling evidence of the activation and proliferation of astrocytes and microglia after seizure-precipitating events [90]. With the increasing understanding of neuroinflammatory processes in epileptic brains, more and more inflammatory mediators such as interleukin and tumor necrosis factors can be used as potential biomarkers to provide valuable information for the diagnosis and prognosis of unpredictable seizures [9].

In a cohort study involving 256 European adults, investigating the correlation between cerebrospinal fluid (CSF) and serum pro-inflammatory cytokine IL-1β levels and the development of post-traumatic epilepsy after moderate-to-severe TBI, multivariate analysis showed that higher CSF/serum IL-1β ratios during the acute phase of injury are associated with an increased risk of post-traumatic epilepsy [91]. Elevated IL-1β was also found in both the epileptic mouse [92] and the epileptic dog [93] models. Similarly, in a study of children with epilepsy aged 4 to 17 years, the investigators also found higher IL-1β in the severe epilepsy group than in the control group [94]. These findings suggest that CSF/serum IL-1β ratios could serve as useful predictors in this context.

Interleukin 6 (IL-6) is a pro-inflammatory cytokine that plays an important role in the modulation of both acute and chronic inflammatory responses [95]. Previous studies have shown that the cerebral overexpression of IL-6 is associated with the progression of seizure-like activity [96]. For instance, the level of IL-6 in the animal model of pilocarpine-induced SE is increased in microglia with the M1 phenotype. Transgenic mice with glial fibrillary acidic protein promoter-driven-overexpression of IL-6 in astrocytes are more sensitive to KA-induced seizures but not to pilocarpine-induced seizures, compared with wild-type mice [97]. These results suggest that a certain basal level of IL-6 is necessary for maintaining the threshold of seizure onset. Monitoring of IL-6 levels helps to understand the progress of epileptogenesis.

Tumor necrosis factor ɑ (TNF-ɑ) is another pro-inflammatory cytokine primarily released by activated microglia and astrocytes in response to neuroinflammatory challenges [98]. Huang *et al*. found that TNF-ɑ-mediated necrotizing apoptosis induces brain endothelial injury, which can be combined with neuroinflammation and astrocyte Kir 4.1 dysregulation, leading to increased susceptibility to seizures in mice [99]. TNF-ɑ-mediated signaling can trigger microglial glutamate release by upregulating glutaminase [100]. Computational modeling of neuron-glia interactions has also provided evidence that TNF-ɑ overexpression leads to seizure-like activity patterns [101]. In humans, TNF-ɑ levels in serum and CSF are higher in epileptic patients than in healthy controls. A higher concentration of TNF-ɑ was associated with higher seizure sensitivity in cohort studies [102]. Monitoring TNF-ɑ levels can be helpful in judging and monitoring epileptogenesis.

### Neuroimaging Biomarkers

Imaging biomarkers have proven to be valuable tools for detecting structural changes associated with epileptogenesis. Magnetic resonance imaging (MRI), positron emission tomography, and single photon emission computed tomography have been extensively applied to study epilepsy-related biomarkers, making it possible to identify the biomarkers associated with the process of epileptogenesis.

The crosstalk among components of the NVU plays a key role in the regulation of blood flow, response to injury, neuronal firing, and synaptic plasticity. BBB dysfunction is a prominent characteristic of brain injury and is often reported in SE [41]. Changes in the BBB have been identified as a sensitive and specific predictor for epilepsy, making it a clinically relevant predictive indicator for acquired epilepsy [103]. MRI is a sensitive method for detecting structural and functional changes in the brain. Previous studies have used gadobutrol-enhanced T1-weighted MRI to pinpoint the location of BBB leakage *via* a thalamic calcification region surrounded by RECA-1 and CD68 in rat models of induced epilepsy [104, 105].

Although an increasing number of studies have screened candidate biomarkers, none of them have confirmed their role in predicting and monitoring the disease state during epileptogenesis. Until now, the study of biomarkers of epilepsy is in a dilemma, and further breakthroughs are urgently needed. Notably, the process of epilepsy is irregular, and biomarkers can only be detected in a specific time window, rather than being continuously monitored throughout the process. Consequently, continuous sampling should be considered to assess the trend of pathophysiological changes in biomarkers. Nevertheless, it is essential to recognize that continuous sampling can lead to high detection costs. As an alternative approach, using different biomarkers at different phases of epileptogenesis may offer advantages in capturing the dynamic nature of the disease progression [9].

## Therapeutic Strategies of Anti-epileptogenesis

Epileptogenesis is defined as the prolonged process of converting a normal mammalian brain into an epileptic brain, characterized by hypersynchronous spontaneous seizures involving a complex epileptic pathogenesis network [106]. The period between brain injury and the onset of epilepsy presents a potential window for intervention, where effective measures can prevent epileptogenesis. Despite the availability of over 30 anti-seizure medications (ASMs) to treat and prevent seizures, none of them can prevent epileptogenesis, thus leading to limited interventions to prevent epilepsy after potential brain injury [107]. Furthermore, even though ASMs can reduce the frequency and severity of seizures, 30% of epileptic patients still develop refractory drug-resistant epilepsy. Consequently, there is a growing recognition of the urgency for preventive treatment before epilepsy seizures occur.

Given the current state of research, shifting the focus from ASM to anti‑epileptogenesis (AEG) strategies holds immense importance. The transformation of this key issue can lead to breakthroughs in preventing epileptogenesis and significantly improve the management of epilepsy in at-risk individuals.

### Anti-epileptogenesis Effects of Antiepileptic Drugs Targeting Neurons

**Fig. 3:**
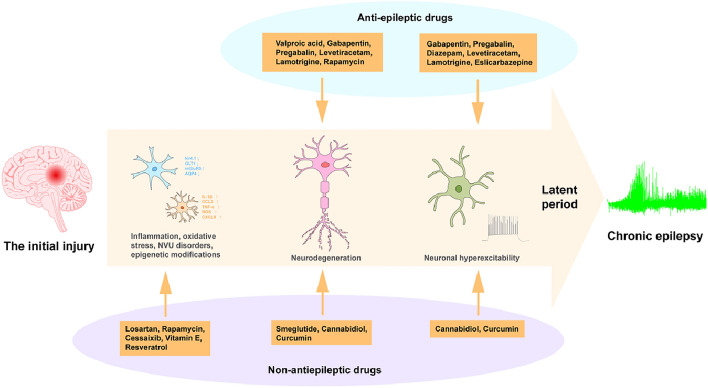
Therapeutic Strategies of Anti-epileptogenesis.

Current available pharmacological treatment of epilepsy mainly focuses on symptom control to manage seizures, but it does not modify the underlying epileptogenesis process [108]. Notably, in specific circumstances such as TBI, the preventive use of anti-epileptic drugs in patients at risk, suffering SE, or with brain infections, can effectively prevent the onset of seizures. For example, certain medications such as valproic acid, gabapentin, pregabalin, diazepam, levetiracetam, and lamotrigine can effectively protect against potential brain injury in the context of SE [109], while others like carbamazepine or phenytoin sodium may not exhibit significant neuroprotection [110] (Fig. 3).

After inducing SE by KA in rats, administering high doses of valproic acid daily for 40 days prevented hippocampal neurodegenerative pathological changes and inhibited spontaneous seizures [111]. Gabapentin (reduces acquired epilepsy) and pregabalin (prolongs the latency of spontaneous convulsions) have shown neuroprotective effects after chemical-induced SE. Diazepam, when given as a high dose after SE, significantly reduced the occurrence of spontaneous seizures evoked by electrical stimulation of the amygdala in rats. Levetiracetam, given at a very high dose (500 mg/kg) after pilocarpine-induced SE in rats, effectively reduced the number of spontaneous seizures and mortality, along with exerting neuroprotective effects [112]. Lamotrigine, a relatively new anti-epileptic drug blocking voltage-operated Na^+^ channels, significantly inhibited spontaneous seizure activity and reduced neurodegeneration and astrogliosis in the hippocampus when administered daily to rats with lithium-pilocarpine-induced SE [113].

Although carbamazepine and phenytoin have been shown to be ineffective in terms of epileptogenesis, the third-generation of the anti-epileptic drug eslicarbazepine, which blocks both Na^+^ channels and T-type voltage-dependent Ca^2+^ channels, has been demonstrated to be an effective inhibitor of epileptogenesis in the pilocarpine model of mouse SE [114]. These findings suggest that specific anti-epileptic drugs may offer the potential for disease-modifying effects in epileptogenesis and merit further investigation for more targeted preventive treatments.

### Anti-epileptogenesis Effects of Non-antiepileptic Drugs Targeting Blood Vessels and Neuroglial Cells

Different mechanisms contribute to epileptic seizures and epileptogenesis, making drugs developed specifically to inhibit seizures not necessarily effective in preventing epileptogenesis [115]. Hence, researchers need to search for potential AEG drugs in both ASMs and other pharmacological targets (Fig. 3).

Losartan, an antihypertensive drug that blocks angiotensin II receptors, has shown promise as an AEG drug. Recent research has shown that losartan reduces the incidence of epilepsy in arterial hypertension and may be neuroprotective over the long term [116]. Similarly, rapamycin, with its neuroprotective effect in the hippocampus and ability to reduce BBB permeability, demonstrates anti-epileptogenic activity in rats with KA-induced SE [117]. Celecoxib, a nonsteroidal anti-inflammatory drug, significantly reduces the frequency and duration of spontaneous seizures in a lithium-pilocarpine-induced SE model in mice by blocking the cyclooxygenase 2 and HMGB1/TLR-4 pathways [118]. Semiglutide, a novel drug for the treatment of type 2 diabetes, not only reduces the severity of seizures but also improves cognitive impairment in a chronic epileptic mouse model induced by pentylenetetrazol [119].

Clinical trials have also shown potential anti-epileptogenic effects of certain drugs. In two cases of refractory epilepsy in children, melatonin co-administration inhibited seizure activity when combined with other drugs [120].

Various compounds such as L-serine, vitamin E, mitochondrial antioxidants, and polyphenolic compounds derived from plants such as cannabidiol, resveratrol, and curcumin, have demonstrated neuroprotective effects [121, 122].

These preclinical and clinical studies provide evidence for the development of novel neuroprotective agents. However, further clinical research is necessary to ensure safe administration to humans and assess the prevention of epilepsy onset without adverse effects.

### Combined Treatments

Currently, both preclinical and some clinical studies have shown promising results in achieving AEG through the combination of certain ASMs or ASMs with other neuroprotective drugs. In these studies, various combinations of two to four drugs were evaluated using a comparable experimental method in the same mouse model of SE [123]. One notable finding is that the combination of levetiracetam with topiramate and levetiracetam with phenobarbital have a highly synergistic effect, leading to significant inhibition of spontaneous seizures in rats after SE. In addition, a combination of levetiracetam with atorvastatin and ceftriaxone has been found to be the most beneficial, as it not only inhibits the incidence of clinical seizures but also reduces the occurrence of electrographic seizures, with rates of 100% and 60%, respectively [124]. These findings highlight the potential of combining ASMs with other neuroprotective drugs as a promising approach for achieving anti-epileptogenesis.

## Future Prospects/Discussion

Neurons, astrocytes, microglia, and vascular endothelial cells all play important roles in epileptogenesis, but their impact may vary in different brain regions due to the heterogeneity. Therefore, studying neural circuits is crucial for a comprehensive understanding of epileptogenesis. At present, most studies on epilepsy-related circuits focus on the temporal lobe, exploring connections like the hypothalamus-anterior nucleus circuit [125], the hippocampal-entorhinal cortex circuit [126], and the nigra-parafascicular circuit [127]. However, the involvement of circuit connections from other brain regions in the development of epilepsy remains unclear and poorly understood. Future research should investigate more deeply into the relationship between neuronal projections in various brain regions to gain valuable insights beyond the immediate epileptic lesions.

Epileptogenesis is a complex process, and not all mechanisms may occur during its course. Identifying general and representative biomarkers as a "gold standard" to detect epileptogenesis should be a priority in current research. While early intervention in the process of epileptogenesis can achieve the purpose of preventing epilepsy, selecting the appropriate time window for treatment is challenging due to the extended duration of the process. By categorizing the stages of epileptogenesis (initial injury, latent period, and chronic period) and identifying specific biomarkers for each stage, combined with drug treatment, better preventive effects may be achieved [2].

Given the intricate nature of epileptogenesis, there are still numerous unresolved issues and challenges. The selection of “gold standard" biomarkers and the complex mechanisms involved in epileptogenesis require further understanding. From an etiological perspective, common factors that can induce epileptogenesis include pediatric febrile seizures [128], neonatal hypoxia [129], and brain injuries [130]. Animal models have played a significant role in understanding the mechanisms and treatment of human epilepsies [131]. Indeed, both acute pharmacological animal models [132] and chronic animal models [133] used in the study of absence epilepsy to gain a better understanding of the condition and to test potential treatments; AY-9944 and MAM-AY are indeed animal models used to study atypical absence seizures [134], and they are valuable tools for researchers working to understand the mechanisms underlying atypical absence seizures and explore potential treatments. The investigation of infantile spasms in animal models is recent and intriguing [135, 136]. Furthermore, it is noteworthy that some of these models have been developed based on the recently recognized pathogenesis of epileptogenesis in various clinical epilepsy syndromes. These syndromes include benign familial neonatal epilepsy, early infantile encephalopathy, severe myoclonic epilepsy of infancy, the tuberous sclerosis model, and progressive myoclonic epilepsy [137–140]. The contribution of animal models to epilepsy research is undeniable. Meanwhile, continuous improvement of existing models and the development of new models are necessary to unveil novel approaches for treating epilepsy patients, with the optimistic goal of enabling clinicians to prevent epilepsy.

In addition, finding effective strategies to intervene in the occurrence of epilepsy using existing drugs during the gap period in new drug development is essential. Addressing these challenges and achieving effective detection and treatment of epilepsy will necessitate further in-depth research.
